# The long non-coding RNA *H19* suppresses carcinogenesis and chemoresistance in hepatocellular carcinoma

**DOI:** 10.15698/cst2017.10.105

**Published:** 2017-08-25

**Authors:** Christina S. Schultheiss, Stephan Laggai, Beate Czepukojc, Usama K. Hussein, Markus List, Ahmad Barghash, Sascha Tierling, Kevan Hosseini, Nicole Golob-Schwarzl, Juliane Pokorny, Nina Hachenthal, Marcel Schulz, Volkhard Helms, Jörn Walter, Vincent Zimmer, Frank Lammert, Rainer M. Bohle, Luisa Dandolo, Johannes Haybaeck, Alexandra K. Kiemer, Sonja M. Kessler

**Affiliations:** 1 Department of Pharmacy, Pharmaceutical Biology, Saarland University, Saarbrücken, Germany.; 2 Faculty of Science, Beni-Suef University, Bani Suwaif, Egypt.; 3 Department for Computational Biology and Applied Algorithmics, Max Planck Institute for Informatics, Saarland Informatics Campus, Saarbrücken, Germany.; 4 School of Electrical Engineering and Information Technology, German Jordanian University, Amman, Jordan.; 5 Department of Genetics and Epigenetics, Saarland University, Saarbrücken, Germany.; 6 Institute of Pathology, Medical University of Graz, Graz, Austria.; 7 Institute of Pathology, Saarland University, Campus Homburg, Homburg (Saar), Germany.; 8 Department of Medicine II, Saarland University Medical Center, Saarland University, Homburg (Saar), Germany.; 9 Cluster of Excellence in Multimodal Computing and Interaction, Saarland Informatics Campus, Saarbrücken, Germany.; 10 Center for Bioinformatics, Saarland University, Saarbrücken, Germany.; 11 Institut Cochin, Inserm U1016, CNRS UMR 8104, Paris, France.; 12 Department of Pathology, Medical Faculty, Otto-von-Guericke University Magdeburg, Magdeburg, Germany.

**Keywords:** loss of imprinting, miR-675, Ki67, ELAVL1/HuR, flow cytometry, SNuPE, Bi-PROF

## Abstract

The long non-coding RNA (lncRNA) *H19* represents a maternally expressed and epigenetically regulated imprinted gene product and is discussed to have either tumor-promoting or tumor-suppressive actions. Recently, *H19* was shown to be regulated under inflammatory conditions. Therefore, aim of this study was to determine the function of *H19* in hepatocellular carcinoma (HCC), an inflammation-associated type of tumor. In four different human HCC patient cohorts *H19* was distinctly downregulated in tumor tissue compared to normal or non-tumorous adjacent tissue. We therefore determined the action of *H19* in three different human hepatoma cell lines (HepG2, Plc/Prf5, and Huh7). Clonogenicity and proliferation assays showed that *H19* overexpression could suppress tumor cell survival and proliferation after treatment with either sorafenib or doxorubicin, suggesting chemosensitizing actions of *H19*. Since HCC displays a highly chemoresistant tumor entity, cell lines resistant to doxorubicin or sorafenib were established. In all six chemoresistant cell lines *H19* expression was significantly downregulated. The promoter methylation of the *H19* gene was significantly different in chemoresistant cell lines compared to their sensitive counterparts. Chemoresistant cells were sensitized after *H19* overexpression by either increasing the cytotoxic action of doxorubicin or decreasing cell proliferation upon sorafenib treatment. An *H19* knockout mouse model (*H19*Δ3) showed increased tumor development and tumor cell proliferation after treatment with the carcinogen diethylnitrosamine (DEN) independent of the reciprocally imprinted insulin-like growth factor 2 (IGF2). In conclusion, *H19* suppresses hepatocarcinogenesis, hepatoma cell growth, and HCC chemoresistance. Thus, mimicking *H19* action might be a potential target to overcome chemoresistance in future HCC therapy.

## INTRODUCTION

Non-coding sequences constitute the considerably larger part of the transcribed human genome compared to coding sequences since only 2% of the genome encode for proteins 1.

Recently, RNA-seq datasets were used to identify long non-coding RNAs (lncRNAs) aberrantly expressed under inflammatory conditions. The well-described lncRNA *H19* (long intergenic non-protein coding RNA 8), a maternally expressed imprinted gene product, was the lncRNA with the most consistent overexpression among all conditions investigated 2. Since cholangiocarcinoma represents a tumor type that develops under inflammatory conditions and in settings of oxidative stress, the authors investigated the role of *H19* in cholangiocarcinoma cell lines and observed tumor-promoting and pro-inflammatory actions of *H19* 2, In contrast, *H19* was found to have tumor-suppressing abilities in colorectal cancer, another inflammation-associated tumor entity 3, and the role of *H19* in inflammation is conflicting 4,5. Embedded in *H19*'s first exon is the microRNA *miR-675* 6, the processing of which is negatively regulated by the mRNA binding protein ELAV like RNA binding protein 1 (ELAVL1 / HuR) 7, and has also been reported to affect cancer 8 and inflammation 4,5,9,10.

Also hepatocellular carcinoma (HCC) evolves in an environment governed by metabolic and inflammatory stress as found in chronic viral hepatitis, as well as in alcoholic and non-alcoholic steatohepatitis 11. HCC represents the second most common cause of cancer-related death worldwide 12, which is not least due to its high chemoresistance. However, the role of *H19* in HCC development, progression, and chemoresistance is still unclear. While Yoshimizu *et al*. reported accelerated tumor development in *H19* knockout mice in SV40-induced HCC 13, *Matouk et al*. observed an enhanced tumorigenic potential of carcinoma cells *in vivo* upon ectopic *H19* expression 14.

Allelic expression of *H19* is controlled by an imprinting control region and by a promoter, which can be differentially methylated 15. Loss of imprinting (LOI), i.e. biallelic *H19* expression, was reported for HCC using small sample size cohorts 16,17. In general, human data on *H19* expression in HCC should be interpreted with caution because the number of samples available for the studies dealing with this topic was mostly rather small 16,17.

We therefore conducted comprehensive studies using four independent patient cohorts, *H19* knockout mice, and three different human hepatoma cell lines to decipher the role of *H19* in HCC development, hepatoma cell growth, and chemoresistance.

## RESULTS 

**Figure 1:**
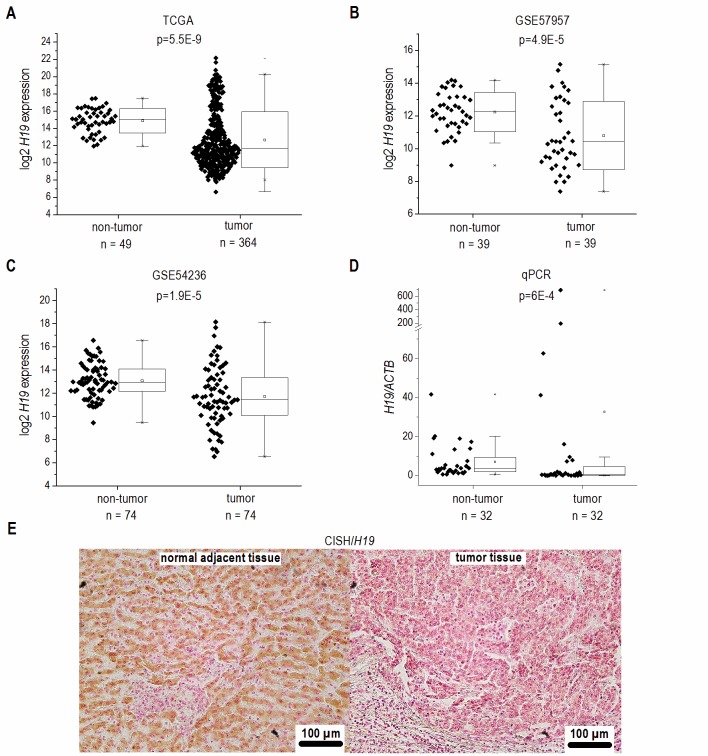
FIGURE 1: *H19* expression in human HCC tissues (tumor) compared to non-tumorous tissues (non-tumor). **(A)** Log2 *H19* expression in HCC tissues from TCGA dataset (non-tumor: n=49, tumor: n=364, Mann-Whitney *U* test). **(B)** Log2 *H19* expression in HCC tissues from GEO dataset GSE57957 (each, n=39, Kolmo- gorov-Smirnov test). **(C)** Log2 *H19* expression in HCC tissues from GEO dataset GSE54236 (each, n=74, Kolmogorov-Smirnov test). **(D)** *H19* expression in HCC tissues from Saarland University Medical Center determined by qPCR (each, n=32, Mann-Whitney *U* test). **(E)** Representative chro- mogenic *in situ* hybridization (CISH) of *H19* (*H19* positive cells: brown; *H19* negative cells: red) (each, n=8).

Based on recent reports suggesting *H19* as an inflammation-inducible lncRNA and HCC representing a disease developing in an inflammatory environment, we sought to determine *H19* expression in human HCC. The comparison of n=364 HCC tissues with n=49 normal liver tissues from TCGA sequencing data revealed highest *H19* expression in a subgroup of HCC samples. Still, statistical analysis of all samples showed an allover decreased expression of *H19* in HCC tissue (**Figure 1A**). Comparing *H19* expression of HCC tissues only to their respective adjacent tissues, *H19* expression was still decreased with high statistical significance (data not shown, p=5.28E-7). Also analysis of two microarray GEO datasets with n=39/39 (GSE57957) and n=74/74 (GSE54236) HCC tissues vs. non-tumor tissue revealed a distinct downregulation of *H19* (**Figure 1B** and **C**) as did qPCR quantification of *H19* in a previously described patient cohort (**Figure 1D**) 18,19. *In situ* hybridization against *H19* revealed low expression of *H19* in tumor tissue, but higher expression in the non-tumorous tissue adjacent to the tumor site in an additional patient cohort (**Figure 1E**). All cohorts comprised patients with HCC from different etiologies. q-PCR of hepatocytes, microdissected from the small subgroup of HCC samples showing high *H19* expression (**Figure 1D**) suggested that *H19* was in fact overexpressed in hepatocytes (Figure S2).

**Figure 2:**
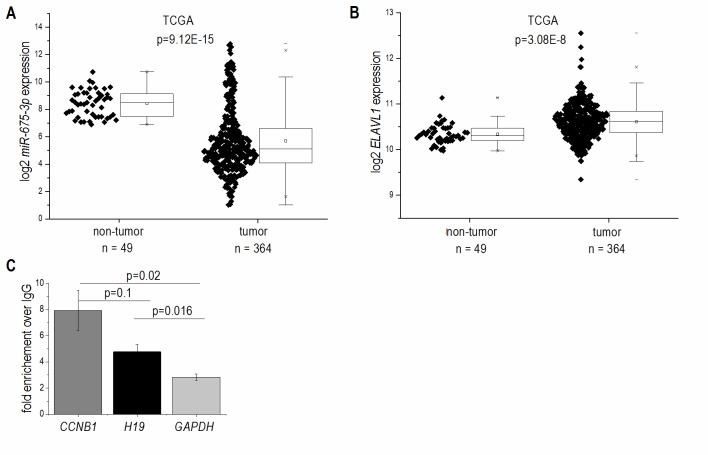
FIGURE 2: *miR-675* and *ELAVL1*/*HuR* expression in human HCC tissues (tumor) compared to non-tumorous tissues (non-tumor). **(A)** Log2 *miR-675-3p* expression and **(B)** Log2 *ELAVL1* expression in HCC tissues from the TCGA dataset (non-tumor: n=49, tumor: n=364, Mann-Whitney *U* test). **(C)** RIP was performed using either IgG or an HuR antibody. Co-precipitated mRNAs *H19*; *CCNB1*, as a positive control; *GAPDH*, as a negative control; were determined by qPCR (n=3, duplicates). Data show x-fold enrichment over the levels found in IgG immunoprecipitates.

In accordance with the results from *H19* expression, which encodes miR-675, the more abundant miR-675-3p was downregulated in HCC (**Figure 2A**) and strongly correlated with *H19* (R²=0.91, p<1.0E-15). The less of the abundant miR-675-5p was not detectable in most samples. The mRNA binding protein HuR/ELAVL1 has been shown to represent a negative regulator of *miR-675* processing in the mouse system by binding to *H19* 7. Interestingly, expression of *ELAVL1* was significantly upregulated in HCC (**Figure 2B**), suggesting an inhibited processing of *H19* into miR-675. RNA immunoprecipitation experiments in Huh7 cells confirmed that HuR/ELAVL1 also binds to human *H19*: *H19* was significantly enriched in HuR immunoprecipitates over the negative control *GAPDH* (**Figure 2C**). Also the postive control *CCNB1* showed a significantly enriched binding compared to the negative control.

The data from independent patient cohorts showed a clear downregulation of *H19* in HCC as a strongly inflammation-associated tumor type. This is why we investigated whether *H19* expression is in fact downregulated due to an inflammatory reaction. In fact, we found a downregulation of *H19* in livers from mice treated with the inflammation-inducing carcinogen DEN (n=5, 0.11 fold ± 0.04 compared to untreated animals, p=0.0508, two-sample t-test).

**Figure 3:**
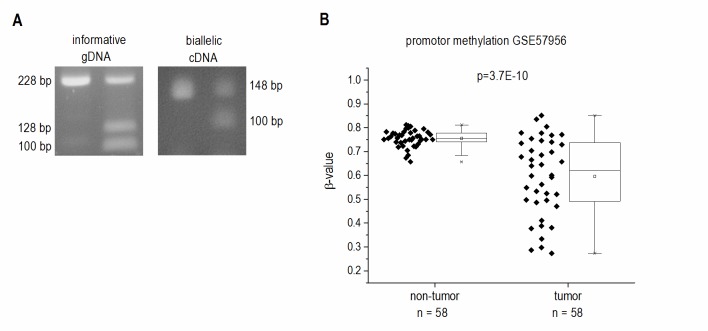
FIGURE 3: Epigenetic state of the *H19* locus in human HCC tissues (tumor) and non-tumorous tissues (non-tumor). **(A)** LOI was analyzed by RFLP analysis of 9 informative gDNA samples. Representative agarose gel with gDNA and cDNA before (left) and after digestion with the restriction enzyme AluI (right). **(B)** *H19* promoter methylation represented as fractional β-values from GEO dataset GSE57956 (each, n=58, Kolmogorov-Smirnov test).

Since *H19*`s expression is epigenetically controlled and LOI of *H19* has been found in some tumor types, we determined allelic expression of *H19* in human HCC by RFLP analysis employing the 32 samples from our patient cohort (**Figure 1D**). The experiment showed that nine of the patients were heterozygous and therefore informative for RFLP analysis (**Figure 3A**). LOI was observed in three normal as well as in three tumor tissues while the other tissues showed monoallelic expression (**Figure 3A**). These findings suggest that LOI is not involved in the deregulation of *H19* expression in HCC.

Besides its regulation by imprinting mechanisms, *H19* expression is also distinctly regulated by the extent of its promoter methylation 20,21. Thus, we analyzed the HCC methylation dataset GSE57956 regarding *H19* promoter methylation. This dataset also comprises the 39 samples, for which *H19* expression was already determined (GSE57957, **Figure 1B**). The analysis revealed a distinctly decreased *H19* promoter methylation with high statistical significance in HCC vs. normal tissues (**Figure 3B**).

**Figure 4:**
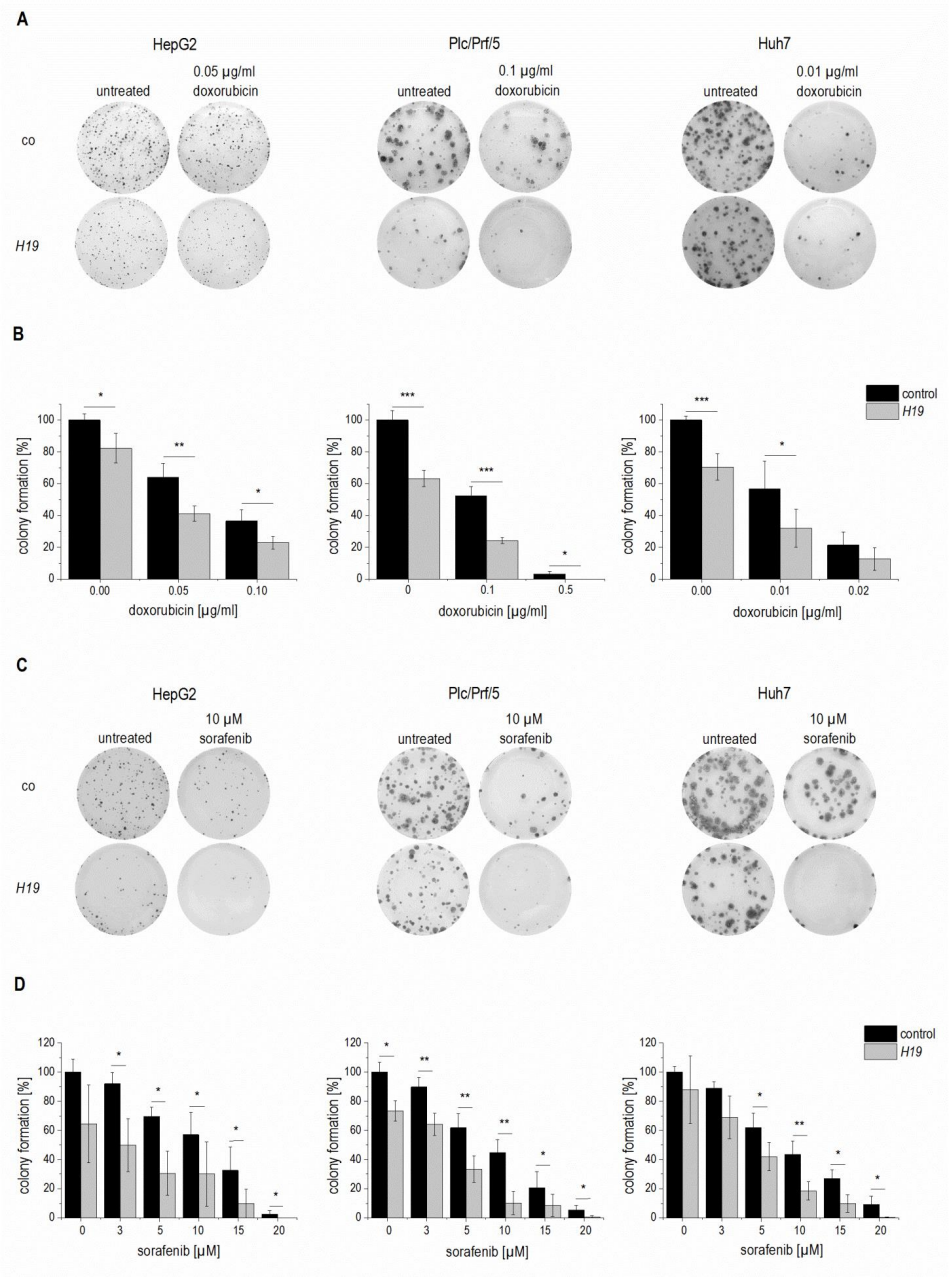
FIGURE 4: Effect of *H19* overexpression on colony formation ability in stably *H19* overexpressing (*H19*) and vector control (control, co) HepG2 (left panels), Plc/Prf/5 (middle panels), and Huh7 (right panels) cells. **(A, C)** Representative results of clonogenicity assays using untreated and **(A)** doxorubicin or **(C)** sorafenib treated hepatoma cells. **(B, D)** Colony formation ability of *H19* overexpressing cells normalized to their respective untreated control cells after **(B)** doxorubicin (n≥3, duplicates) or **(D)** sorafenib (n=3, triplicates) treatment. The p values were calculated by two-sample t-test or Mann-Whitney *U* test depending on the data distribution. * p < 0.05, ** p < 0.01, *** p < 0.001.

Due to the downregulation of *H19* in HCC we aimed to determine functional aspects of *H19* overexpression in liver cancer cells. Thus, the colony formation assay - a well established method to determine every cell’s ability to undergo unlimited division in a cell population 22 - was performed in three different stably *H19* overexpressing human hepatoma cell lines. All three cell lines we investigated, i.e. HepG2, Plc/Prf/5, and Huh7, showed that *H19* suppresses tumor cell survival, as indicated by a reduced colony number (**Figure 4A-D**).

To explore the potential role of *H19* in chemosensitivity, the three stably *H19* overexpressing cell lines were treated with either sorafenib or doxorubicin, two therapeutics which have clinically been tested for HCC treatment 23,24. All stably *H19* overexpressing cell lines showed significantly increased sensitivity in the clonogenicity assay, suggesting a chemotherapy-sensitizing action of *H19* (**Figure 4A-D**). In order to distinguish reduced colony formation from chemosensitizing actions of *H19*, we also performed a different data normalization strategy, which can be found as supplemental Figure S3. Also this quantification confirmed a chemosensitizing action of *H19*.

*miR-675* is unlikely to be responsible for this action: while *H19* expression was significantly upregulated up to 65-fold ± 9.4 in stably transfected cells (n=3, triplicates, p=2.4E-6, two-sample t-test), two of the three cell lines showed no increase in *miR-675* expression (n=3, triplicates, each: HepG2: 9.2-fold ± 7.3, p=0.06, Mann-Whitney *U*; Huh7: 1.7-fold ± 1.0, p=0.36, Mann-Whitney *U*). Only stably *H19* overexpressing Plc/Prf/5 cells revealed slightly upregulated *miR-675* expression (n=3, triplicates, 1.5-fold ± 0.17, p=1.2E-3, two-sample t-test), while *H19* was 6.9-fold higher expressed (n=3, triplicates, 10.3-fold ± 2.3, p=1E-4). *ELAVL1* mRNA levels were not affected in all three cell lines upon *H19* overexpression (data not shown). In HepG2 and Huh7, the action was independent of the anti-apoptotic growth factor IGF2 18, frequently regulated in parallel with *H19* due to the genomic vicinity and shared imprinting control region 25: *IGF2* expression was unchanged in both stably *H19* overexpressing cell lines compared to empty vector-transfected controls (n=3, triplicates each: HepG2: 1.4-fold ± 0.6, p=0.16, Mann-Whitney *U* test; Huh7: 1.1-fold ± 0.1, p=0.17, two-sample t-test). Still, in Plc/Prf/5 the expression of *IGF2 *was significantly downregulated (n=3, triplicates,0.3-fold ± 0.1, p=3.9E-6, two-sample t-test). Interestingly, Plc/Prf/5 exhibited a highly increased intrinsic chemoresistance compared to the other two cell lines.

**Figure 5:**
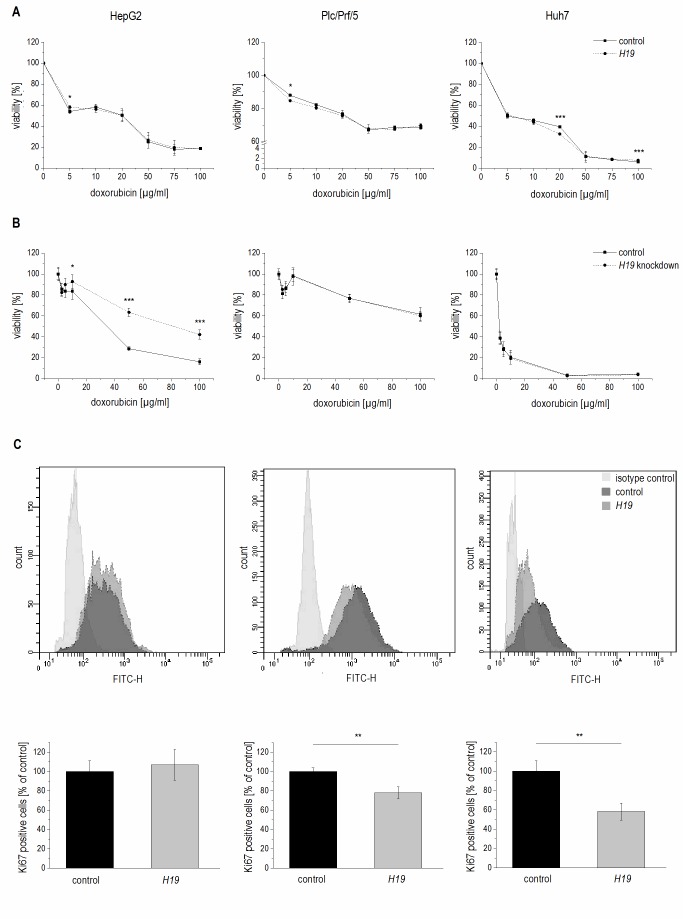
FIGURE 5: Effect of *H19* overexpression and knockdown on cell viability and proliferation in HepG2 (left panels), Plc/Prf/5 (middle panels), and Huh7 (right panels) cells. **(A)** Cytotoxicity assay with doxorubicin in stably *H19* overexpressing (*H19*) or vector control (control) cells normalized to their respective untreated control (n=2, sextuplicates). **(B)** Cytotoxicity assay with doxorubicin after transfection with *H19* gapmer (*H19* knockdown) and control gapmer (control) normalized to their respective untreated control (n=2, sextuplicates). **(C)** FACS analysis of the proliferation marker Ki67 in stably *H19* overexpressing (*H19*) and vector control cells (control). Representative histograms of Ki67 FACS analysis are shown (upper panels). Quantification of Ki67 positive cells expressed as percent of control (n≥2, triplicates). The p values were calculated by two-sample t-test or Mann-Whitney *U* test depending on the data distribution. * p < 0.05, ** p < 0.01, *** p < 0.001.

In order to determine whether *H19* overexpression or knockdown directly affected cytotoxicity, cell viability was measured by MTT assay either in stably *H19* overexpressing cells or in cells with a gapmer-facilitated *H19* knockdown upon treatment with the cytotoxic agent doxorubicin. Cell viability with overexpressed or knocked down *H19* was largely unchanged in Plc/Prf/5 and Huh7 cells, although a few values reached statistical significance (**Figure 5A** and **B**). Only in *H19* gapmer-treated HepG2 cell viability was distinctly elevated compared to gapmer control cells (**Figure 5B**). These heterogenous findings suggested that *H19*-facilitated chemosensitization in Plc/Prf/5 and Huh7 is unlikely to depend on altered cell death but might rather depend on reduced proliferative capacity.

Therefore, proliferation measurements in *H19* overexpressing cells by Ki67 staining and subsequent FACS quantification were performed. Interestingly, while proliferation of HepG2 cells was unchanged in *H19* overexpressing cells, *H19* exhibited a significant proliferation-suppressing activity in Plc/Prf/5 and Huh7 cells (**Figure 5C**).

**Figure 6:**
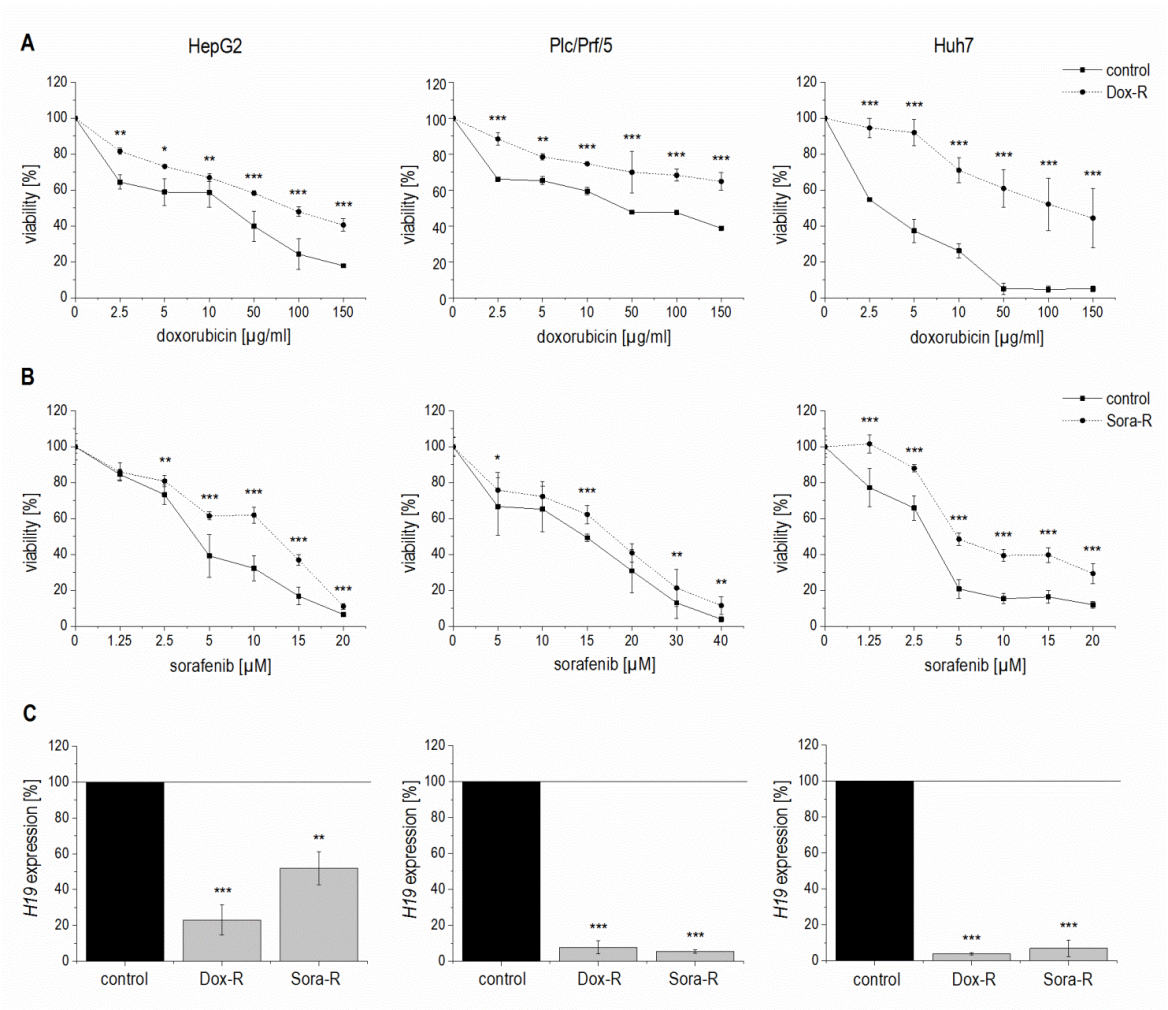
FIGURE 6: Validation of chemoresistance and expression of *H19* in sorafenib resistant (Sora-R), doxorubicin resistant (Dox-R), and chemosensitive (control) HepG2 (left panels), Plc/Prf/5 (middle panels), and Huh7 (right panels) cells. **(A, B)** Cytotoxicity assay normalized to the respective untreated control in **(A)** doxorubicin resistant cells (n=2, quintuplicates) and **(B)** sorafenib resistant cells (n=3, quintuplicates). **(C)** *H19* expression determined by qPCR in doxorubicin (n=3, duplicates) and sorafenib (n=3, triplicates) resistant cells normalized to control cells. The p values were calculated by two-sample t-test or Mann-Whitney *U* test depending on the data distribution. * p < 0.05, ** p < 0.01, *** p < 0.001.

We hypothesized that a downregulation of *H19* might also contribute to chemoresistance as induced by repeated treatment with chemotherapeutics. To test this hypothesis, we established doxorubicin- and sorafenib-resistant HepG2, Plc/Prf/5, and Huh7 cell lines by repeated treatment with the drugs. Their chemoresistance was confirmed by directly comparing their sensitivity with non-resistant cells towards the drugs in a dose-response analysis (**Figure 6A** and **B**).

Quantifying *H19* expression by qPCR revealed that chemoresistance was associated with strongly downregulated *H19* expression in doxorubicin resistant cells (**Figure 6C**). However, *miR-675* was not significantly affected in any of the doxorubicin resistant cells (n=2, duplicates, each: HepG2-Dox-R: 0.52-fold ± 0.30, p=0.11, two-sample t-test; Plc/Prf/5-Dox-R: 0.71-fold ± 0.25, p=0.20, two-sample t-test; Huh7-Dox-R: 2.13-fold ± 0.86 p=0.16, two-sample t-test). Also in sorafenib resistant cell lines *H19* expression was significantly suppressed (**Figure 6C**).

Analysis of the *multidrug resistance protein 1* (*MDR1*, *ABCB1*) showed increased expression in all doxorubicin (n=2, duplicates, each: HepG2-Dox-R: 44.2-fold ± 8.8, p=6.2E-3, two-sample t-test; Plc/Prf/5-Dox-R: 1.8-fold ± 0.2, p=3.1E-2, Mann Whitney *U* test; Huh7-Dox-R: 6.7-fold ± 0.4, p=2.3E-4, two-sample t-test) and sorafenib resistant (n=3, triplicates, each: HepG2-Sora-R: 4.1-fold ± 0.7, p=4.5E-5, two-sample t-test; Huh7-Sora-R: 1.5-fold ± 0.2, p=3.4E-4, two-sample t-test) cell lines except for sorafenib resistant Plc/Prf/5 cells (n=3, triplicates, Plc/Prf/5-Sora-R: 0.6-fold ± 0.1, p=2.8E-6, two-sample t-test).

**Figure 7:**
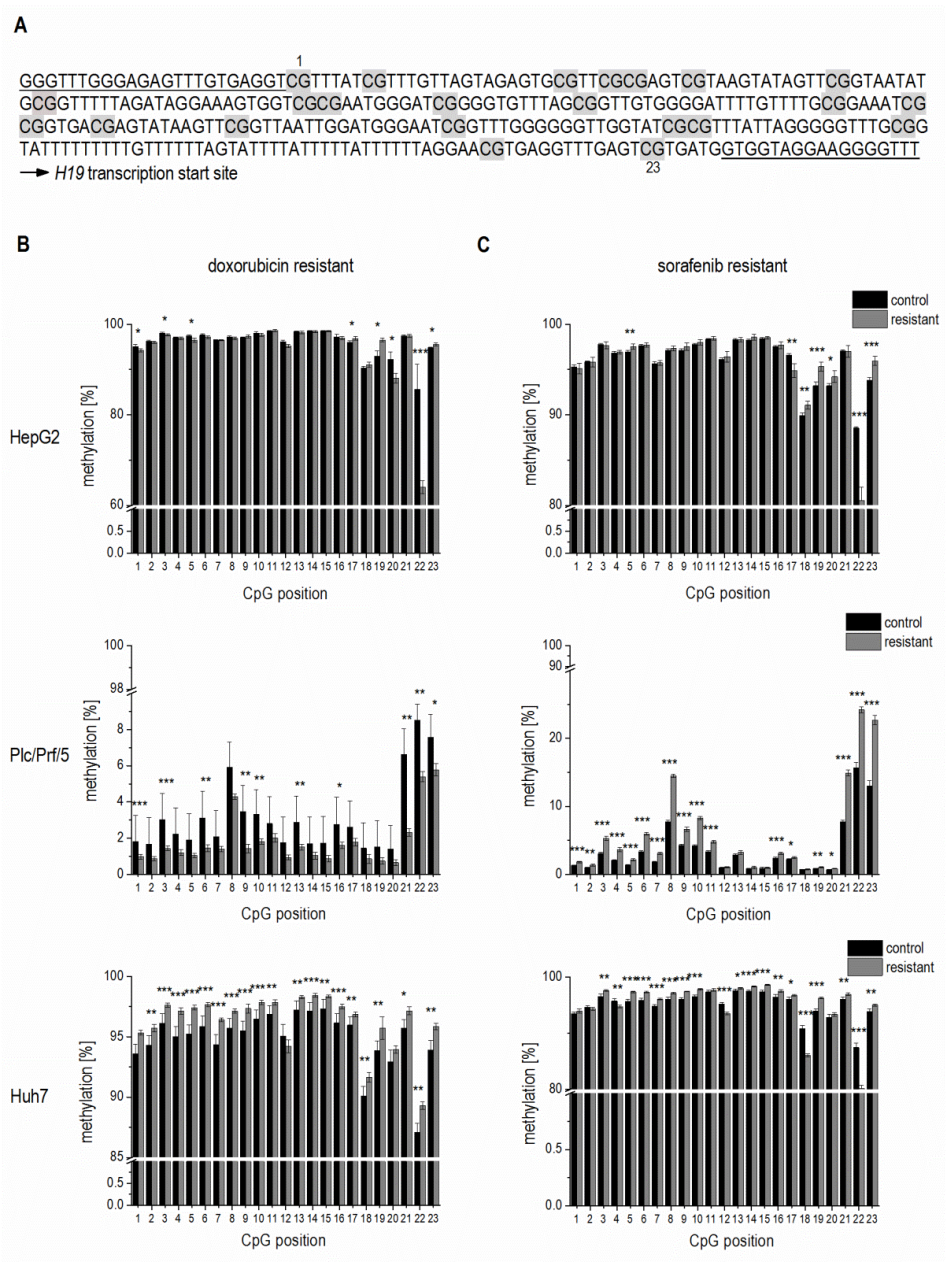
FIGURE 7*: *Methy- lation state of the *H19* promoter in chemoresistant cells. **(A)** Analyzed *H19* promoter region. The bisulphite-converted DNA sequence is shown; CpG sites 1-23 (Chr.11: 2,019,761 - 2,019,488) are la- belled in grey; amplification primers are underlined. **(B, C)** Absolute methylation of 23 CpG sites located in the *H19* promoter in chemo-resistent (resistant) and chemosensitive (control) HepG2 (up- per panels), Plc/Prf/5 (middle panels), and Huh7 (bottom panels) cells analyzed by Bi-PROF (n=3, triplicates). **(B)** Doxorubicin resistant and **(C)** sorafenib resistant hepatoma cells. The p values were calculated by two-sample t-test or Mann-Whitney *U* test depending on the data distribution. * p < 0.05, ** p < 0.01, *** p < 0.001.

To elucidate if altered promoter methylation is again linked to changed *H19* expression, the *H19* promoter methylation status was analyzed by local deep bisulfite sequencing (Bi-PROF) covering 23 CpG sites (**Figure 7A**) in the six chemoresistant cell lines. All three cell lines resistant for sorafenib showed elevated CpG methylation compared to their sensitive counterparts (**Figure 7C**): in Plc/Prf/5 cells 18 out of 23 investigated CpGs showed an elevated methylation; in HepG2 only 5 CpGs were hypermethylated (with 2 hypomethylated), while most CpGs were hypermethylated in Huh7 cells. Almost all investigated CpGs in Huh7 also showed an elevated methylation in doxorubicin resistance. Interestingly, though, almost half of the CpGs were hypomethylated in doxorubicin resistant Plc/Prf/5 cells, and an almost equal number of CpGs was hyper- or hypomethylated in doxorubicin resistant HepG2 (3 up, 4 down) (**Figure 7B**). Taken together, although only Huh7 cells showed a consistent distinct hypermethylation, all three cell lines altered the methylation state of the *H19* promoter during chemoresistance. Most differences were found at CpG sites close to the transcription start site of *H19* (**Figure 7A-C**). Since these findings were highly reproducible in three independent biological replicates and similar to deregulated promoter methylation in HCC samples, we suggested an involvement of a deregulated promoter methylation in suppressed *H19* expression during chemoresistance.

**Figure 8:**
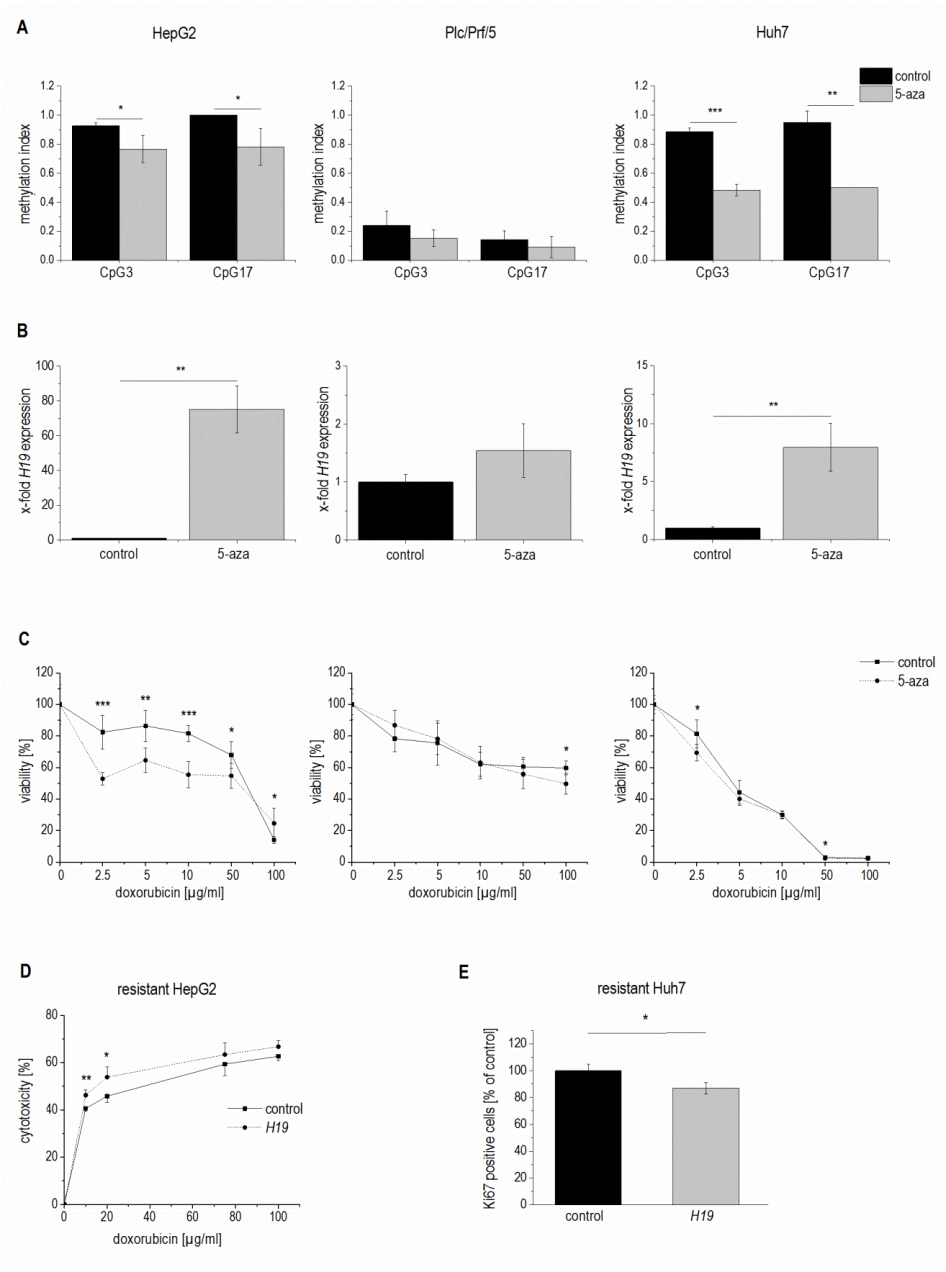
FIGURE 8: Methylation dependent *H19* expression and its effect on cell viability and proliferation. **(A-C)** HepG2 (left panel), Plc/Prf/5 (middle panel), and Huh7 cells (right panel) treated with 5-azacytidine (5-aza) and untreated control cells (control) were analyzed for **(A)** methylation index of two CpG sites of the *H19* promoter by SNuPE (n=2, duplicates), **(B)** *H19* expression determined by qPCR (n=2, duplicates), and **(C)** viability after treatment with doxorubicin by cytotoxicity assay (n=2, quintuplicates). **(D)** Cytotoxicity estimated by MTT assay after treatment with doxorubicin in doxorubicin resistant HepG2 either transiently overexpressing *H19* (*H19) *or vector control transfected (control) for 48 h normalized to the respective untreated control (n=2, triplicates). **(E)** Quantification of Ki67 positive cells by FACS in sorafenib resistant Huh7 either transiently overexpressing *H19* (*H19)* or vector control transfected (control) for 48 h and expressed as percent of control (each, n≥2, duplicates). The p values were calculated by two-sample t-test or Mann-Whitney *U* test depending on the data distribution. * p < 0.05, ** p < 0.01, *** p < 0.001.

Therefore, we tested whether 5-azacytidine, a DNA demethylating agent, had an effect on chemosensitivity. As expected, the compound altered the CpG methylation of the *H19* promoter in two of the three cell lines as measured by SNuPE (**Figure 8A**) and significantly increased *H19* expression in the same two cell lines (**Figure 8B**) with the strongest effect being seen in HepG2 cells, which were also distinctly sensitized towards doxorubicin in the presence of 5-azacytidine (**Figure 8C**). This is why HepG2 cells were also employed for an approach to test whether *H19* overexpression can reverse chemoresistance. We in fact observed an increased induction of cell death by doxorubicin after transfecting chemoresistant cells with *H19* (**Figure 8D**). Since *H19* rather seemed to act on proliferative actions in Huh7, we overexpressed *H19* in chemoresistant Huh7 cells and in fact also observed chemosensitization (**Figure 8E**).

**Figure 9:**
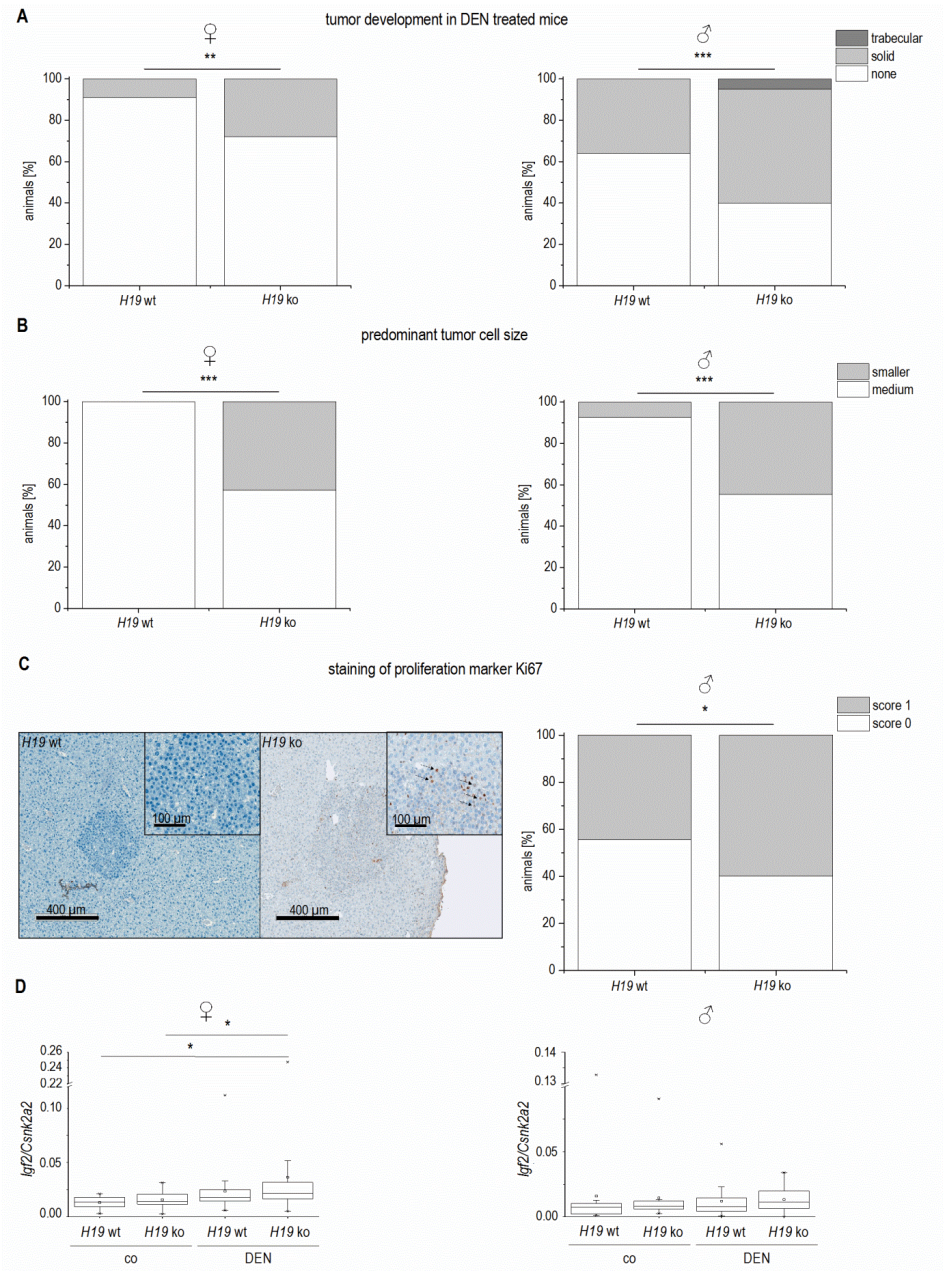
FIGURE 9: Tumor development and characterization in long-term DEN treated *H19* knockout (*H19* ko) compared to *H19* wild-type (*H19* wt) mice. **(A)** Tumor development in female (left) and male (right) DEN treated mice (female: *H19* wt n=22, *H19* ko n=25; male:* H19* wt n=25, *H19* ko n=20). **(B)** Predominant cell size in tumors of DEN treated female (left) and male (right) mice. **(C)** Representative immunohistological staining of proliferation marker Ki67 (left, score 0: no proliferating cells detectable; score 1: less than 1% proliferating cells with brown stained nuclei (arrow)) and expression of Ki67 in tumors of male DEN treated mice (right, *H19* wt n=9, *H19* ko n=10). **(A-C)** The p values were calculated by Chi-square test. * p < 0.05, ** p < 0.01, *** p < 0.001. **(D)** *Igf2* expression in female (left) and male (right) control (co) and DEN treated (DEN) mice determined by qPCR (female: *H19* wt co n=11, *H19* ko co n=23, *H19* wt DEN n=22, *H19* ko DEN n=25; male* H19* wt co n=13, *H19* ko co n=18, *H19* wt DEN n=25, *H19* ko DEN n=20). The p values were calculated by Mann-Whitney *U *test. * p < 0.0125.

The data on reduced expression of *H19* in human HCC and its chemosensitizing actions suggested tumor-suppressive actions of *H19* in HCC. To determine whether the presence of *H19* has an impact on tumorigenesis, wild-type and *H19* knockout animals were treated with the carcinogen DEN for 24 weeks. As expected 26, male mice developed more tumors than female mice (**Figure 9A**). In both sexes, *H19* knockout significantly increased the number of solid tumors. Trabecular tumors were only detectable in male *H19* knockout mice. The histological analysis also indicated that tumors of DEN-treated *H19* deficient mice were characterized by small cell changes, representing dysplastic lesions found in the process of liver carcinogenesis (**Figure 9B**).

The proliferation-suppressive actions of *H19*, as shown in the two human hepatoma cell lines Huh7 and Plc/Prf/5, could also be verified *in vivo*: *H19* deficient long-term DEN-treated animals exhibited elevated Ki67 staining (**Figure 9C**). The expression of the oncogenic growth factor Igf2 was not different between wild-type and *H19* knockout mice (**Figure 9D**), although *Igf2* expression was significantly induced upon DEN treatment in *H19* knockouts (**Figure 9D**).

## DISCUSSION

*H19* was first described more than thirty years ago, at a time when the biological role of non-coding RNAs was still undefined 27. Since *H19* together with *Igf2* and *Igf2r* belongs to the first imprinted genes described 28,29, most of the first two decades of *H19* research focused on its epigenetic regulation.

While mouse data show a distinct downregulation of *H19* expression in all tissues except for skeletal muscle after birth 28, analysis of human samples shows well detectable *H19* levels in a wide array of tissue types (see e.g. http://medicalgenome.kribb.re.kr/GENT. In fact, our analyses of four different patient cohorts comprising several hundreds of samples showed significantly higher *H19 *expression in normal liver tissue compared to HCC tissue. These findings support the findings of a previous study that analyzed *H19* expression in 33 HCC tissues by qPCR compared to either adjacent non-tumor tissue or remote relative normal tissue 30. As in our results, some HCC tissues of the latter study showed a dramatic increase in *H19* expression, but alltogether *H19* was downregulated in HCC with high statistical significance. In fact, *H19* expression was significantly lower in invasive HCC samples (n=31) compared to non-invasive HCC tumors (n=41) 30.

Interestingly, other studies reported elevated *H19* expression in HCC, e.g. 17,31,32,33. It has to be noted, however, that these studies investigated a considerably lower number of samples and/or used methods, such as *in situ* hybridization with limited quantitative reliability. Taking into account that all of our four patient cohorts, as well as those investigated by Zhang *et al*. (2013) 30, contained a small HCC patient subcohort with very high *H19* levels, it becomes clear that investigations of small patient cohorts can lead to contradictory findings.

Also reports on a potential loss of imprinting (LOI), i.e. biallelic *H19* expression, are of limited significance. In fact, some studies have reported LOI in a subset of HCC tissues ranging between 21% and 66%. The sample numbers of these studies, again, were very low (n=3 or n=23) 16,17. Our data revealed no difference in the imprinting status between normal and HCC tissue, with an equal proportion of samples exhibiting biallelic expression.

With the knowledge on a distinct regulation of *H19* expression by epigenetic modifications, we focused on potential alterations in the methylation state of the *H19* promoter. Indeed, we observed strongly altered *H19* promoter methylation in human HCC vs. normal liver tissue. While decreased promoter methylation is typically thought to be linked to elevated gene expression 34,35,36, a hypermethylated promoter region has also been associated with increased expression of some genes 37,38. In fact, our data from a large HCC patient cohort suggested reduced promoter methylation correlating with reduced gene expression. Since HCC and other cancer types are associated with global DNA hypomethylation 39,40,41, the link between *H19* expression and promoter methylation remains unclear, and should be clarified in further studies. We observed the same in the investigated doxorubicin resistant Plc/Prf/5 cell line, which showed both reduced *H19* expression and reduced promoter methylation compared to its chemosensitive counterpart. Interestingly, Plc/Prf/5 showed a much lower baseline promoter methylation compared to both HepG2 and Huh7, suggesting a differential epigenetic profile. Along this line, treatment of Plc/Prf/5 cells with the DNA demethylating agent 5-azacytidine neither affected *H19* promoter methylation nor *H19* expression.

All three sorafenib resistant cell lines as well as doxorubicin resistant Huh7 cells exhibited significantly elevated *H19* promoter methylation and at the same time significantly reduced *H19* expression compared to their respective chemosensitive counterparts. These findings are in line with anti-correlated methylation of the *H19* promoter and expression of *H19* as found by others 20,21. Accordingly, treatment with the methyltransferase inhibitor 5-azacytidine reduced *H19* promoter methylation in both HepG2 and Huh7 cells and significantly increased *H19* expression, as previously observed in other cell types 42. Interestingly, *H19* itself has been reported to increase DNMT3B-mediated cytosine methylation 43, suggesting diverse feedback processes.

Induction of *H19* by 5-azacytidine in HepG2 and Huh7 cells increased their chemosensitivity. This confirms findings in the literature on chemosensitizing actions of the compound 44. Chemoresistant versions of all three investigated cell lines showed downregulated *H19* expression. This suggested chemosensitizing actions of *H19*. In fact, *H19* overexpression sensitized all three tested cell lines against both sorafenib and doxorubicin in clonogenicity assays. This effect seems to result from a synergistic effect of *H19*’s antiproliferative and chemosensitizing actions. These findings on chemosensitizing action of *H19* are in contrast to a paper reporting chemoresistance induction by *H19* and linking it to induction of the multidrug resistance protein (*MDR1*, *ABCB1*) 45. Our investigations employing three different hepatoma cell lines could not verify these effects.

Chemosensitizing actions of *H19* in our hands seemed to differ between the different cell lines: in HepG2 cells, modulation of *H19* expression rather affected cell death, whereas in Huh7 cells, *H19* suppressed proliferation.

Our clonogenicity data suggest growth-suppressive actions of *H19* in the absence of any drug treatment. These data corroborate a hypothesis on the role of maternally expressed genes in embryonic development, already formulated in the early 1990s 46. It provided a model of parental conflict, in which the females, through maternally expressed genes, balance resources allocated to current and future offspring. This notion led to the anticipation that maternally expressed genes limit growth.

Our *in vivo* data employing *H19* knockout mice showed accelerated tumor development and more aggressive tumors. The literature contains two different *H19* knockout mouse models. One of them, the *H19*Δ13 mouse, shows a distinct overgrowth phenotype 47. This overgrowth is facilitated by a full re-expression of the adjacent *Igf2* gene from the normally silent maternal allele due to the combined 13 kb deletion of the *H19* gene and of the imprinting control region 47. In the *H19*Δ3 knockout model, which we used and which only carries a 3 kb deletion of the *H19* gene, only a slight re-expression of the maternal *Igf2* was detected in mesodermal tissue 48. With liver representing an endodermal tissue, *Igf2* expression is not increased in *H19*Δ3 mice as previously reported 48 and confirmed by ourselves.

In contrast to our findings Matouk *et al*. (2007) 14 suggested tumor promoting actions of *H19* in a xenograft model employing Hep3B cells. Different aspects might be responsible for this discrepancy. While our model involves *in vivo* tumor induction, a xenograft model employs established tumor cells. So tumor-inducing actions cannot be investigated with a xenograft model. Another aspect relates to the role of the immune defense, since tumor growth and development are strongly controlled by the immune system and xenograft models employ immune deficient mice. In 2008, Yoshimizu and colleagues investigated the role of *H19* and reported in fact that *in vivo* HCC development was accelerated in *H19* knockout mice 13.

There are several papers that regard *H19* as a promoter of cancer initiation and progression in a set of tumor types 8. Interestingly, most of the *H19* actions in this context have been explained by *miR-675*, a microRNA embedded within *H19*. This microRNA targets a whole array of transcripts, such as *Igf1r*, *Smad1*, *Smad5*, *Cdc6*, *CDH-11* and -*13*, *RB1*, *RUNX1*, *NOMO1*, *TGFBI*, *CALN1*, and *MITF* (reviewed in 8) and promotes cell proliferation 49. In contrast, a recently published paper clearly showed that *H19* reduces proliferation 50.

Taken together, the discrepancy of effects of *H19* in the three different cell lines and the controversial literature data suggest a strong context dependency, and needs to be addressed in further studies.

While the expression of *H19* was always strongly affected in our chemoresistance and proliferation studies, *miR-675* showed minimal expression alterations. This is why we assume that in HCC it is rather *H19* than *miR-675*, which exerts biological actions. Interestingly, the mRNA binding protein HuR/ELAVL1, which has immunoregulatory potential (e.g. 51) and suppresses the processing of *H19 *into *miR-675* 7, is overexpressed in human HCC 52,53, but not differentially expressed in our chemoresistant cell lines. In fact, tumor-promoting actions by HuR/ELAVL1 via the inhibition of microRNA processing in HCC have recently been reported 54.

Taken together, despite a small patient subcohort showing overexpressed *H19*, the majority of HCC tissues contained significantly reduced levels of this epigenetically regulated lncRNA. With its effect on HCC cancer cell growth, chemosensitivity, and carcinogenesis, *H19* shows tumor-suppressive actions. Restoring *H19* actions might therefore represent an interesting approach for future HCC therapy.

## MATERIALS AND METHODS

### Bioinformatic analyses

#### TCGA data

RNAseq expression data were obtained from The Cancer Genome Atlas (https://cancergenome.nih.gov) via the Genomic Data Commons (https://gdc.cancer.gov), using the TCGAbiolinks R package (v.2.2.6) 55. The dataset comprised 364 primary solid tumor as well as 49 matched healthy liver tissue samples. For gene expression analysis, RSEM 56 normalized read counts were downloaded and log2-transformed.

#### GEO datasets

For differential gene expression analyses, 39 tumor and 39 adjacent non-tumor samples from the Total RNA Illumina HumanHT-12 V4.0 dataset GSE57957 were included. Similarly, differential gene expression was analyzed in dataset GSE54236 between tumor (n=74) and non-tumor (n=74) samples of an Agilent-014850 Whole Human Genome Microarray 4x44K G4112F (three samples without sufficient information per group were excluded). Additionally, the methylation of the *H19* promoter region 2 kb around the transcription start site was analyzed using the GSE57956 dataset of bisulfite converted DNA from 58 tumors and 58 adjacent non-tumor samples hybridized to an Illumina Infinium 27k Human Methylation Beadchip (two samples without sufficient information per group were excluded). A subset of samples from GSE57956 was equal to the expression dataset GSE57957.

### Clinical samples

32 human paraffin-embedded liver samples of tumor and matched non-tumorous adjacent tissue from randomly selected pseudonymized HCC patients who underwent liver resection at the Saarland University Medical Center between 2005 and 2010 were obtained 18. The study protocol was approved by the local Ethics Committee (Kenn-Nr. 47/07). Clinical data were described previously 18,19.

### Microdissection and RT-PCR

Tissue sections of paraffin-embedded (FFPE) tissues were mounted on nuclease and human nucleic acid free glass MembraneSlides (Leica Microsystems CMS, Wetzlar, Germany, order no. 11505189), deparaffinized and stained with haemalaun. Laser microdissection was performed as described previously 57,58 using a Leica LMD6000 microscope (Leica Microsystems CMS). Laser-microdissected cells were transferred into a reaction tube containing PKD buffer (Qiagen, Germany). RNA was isolated according to manufacturer’s protocol "Purification of total RNA from microdissected FFPE tissue sections" using the RNeasy FFPE Kit (Qiagen, Hilden, Germany). 28 ng RNA were reverse transcribed using random primers as described 18.

### RNA isolation and quantitative real-time RT-PCR (qPCR)

Isolation of total RNA with QIAzol lysis reagent (Qiagen, Hilden, Germany), DNase digestion, reverse transcription of 0.5 µg RNA, and real-time RT-PCR were performed as described previously . For microRNAs the procedure was performed accordingly with minor modifications: the RNA after isopropanol precipitation was not washed with ethanol and reverse transcription with 2 µg RNA was performed using the miScript II RT Kit and HiSpec Buffer (Qiagen, Hilden, Germany) as recommended by the manufacturer. Samples along with plasmid standard dilution series from 80 to 0.00008 attomol were run in triplicates using 5xHOT FIREPolTM Evagreen® qPCR Mix Plus (Solis BioDyne, Tartu, Estonia) in a CFX96 cycler (Bio-Rad, Munich, Germany) with specific primers (Table S1) (Eurofins Genomics, Ebersberg, Germany). The reaction conditions for the detection of mRNAs were 95°C for 15 min followed by 40 cycles of 30 s at 94°C, 30 s at primer-specific annealing temperature (AT listed in Table S1), and 30 s at 72°C. A melting curve from 55°C to 95°C was recorded to detect potential unintended products. For the detection of microRNAs the reaction conditions were 95°C for 15 min, followed by 40 cycles of 15 s at 94°C, 30 s at primer-specific annealing temperature (AT listed in Table S1), and 30 s at 70°C. The human gene expression samples were normalized to actin beta (ACTB); murine samples to casein kinase 2 alpha 2 (Csnk2a2), and microRNAs were normalized to RNU6B.

### *H19* RNA immunoprecipitation

Immunoprecipitation (IP) of HuR-associated RNAs from Huh7 cells, validation of IP by western blot, and qPCR for the negative control GAPDH and the positive control cyclin B1 were performed as described previously 51. Primer sequences and conditions for *H19* IP qPCR (H19 IP) can be found in supplemental Table S1.

### RFLP (restriction fragment length polymorphism) analysis

Genomic DNA (gDNA) was isolated from paraffin-embedded tissues using the QIAamp DNA FFPE Tissue Kit (Qiagen, Hilden, Germany) according to the manufacturer’s instructions. Genomic DNA was amplified to screen liver tissue samples for heterozygosity at a known AluI polymorphism at the *H19* gene 63. 9 of the 32 samples 18 were heterozygous and therefore corresponding cDNA was tested for biallelic expression. Primer sequences used were the following: 5’-TACAACCACTGCACTACCTG-3’ (sense), 5’-TGGCCATGAAGATGGAGTCG-3’ (antisense). The PCR reaction was performed using the DyNAmo Flash SYBR® Green Master mix containing 400 nM primer, each. Amplification was performed in a Thermal Cycler (Px2 Thermal Cycler, Thermo Electron Corporation, Schwerte, Germany). PCR products were digested for 2 h at 37°C with AluI (Fermentas, St. Leon-Rot, Germany). Detection of existence of polymorphisms and expression status was done by agarose gel electrophoresis showing three bands (228 bp, 128 bp, and 100 bp) in gDNA after digestion. In case of monoallelic expression, cDNA was expected to show one 148 bp band and three bands (48 bp, 100 bp, and 148 bp) in case of biallelic expression. Due to the high agarose concentration (3%), the 48 bp band was not detectable.

### Cell culture

HepG2, Huh7, and Plc/Prf/5 cells were cultured in RPMI-1640 medium with 10% fetal calf serum, 1% penicillin/streptomycin, and 1% glutamine (Sigma-Aldrich, Taufkirchen, Germany) at 37°C and 5% CO2.

#### Establishment of chemoresistant cells

Doxorubicin-resistant (HepG2-Dox-R, Huh7-Dox-R, and Plc/Prf/5-Dox-R) and sorafenib-resistant (HepG2-Sora-R, Huh7-Sora-R, and Plc/Prf/5-Sora-R) cells were established by treatment with increasing concentrations of the cytostatic drugs over several months. In order to maintain the resistance, cells were treated biweekly with doxorubicin (HepG2-Dox-R: 1 µg/ml, Huh7-Dox-R: 0.2 µg/ml, and Plc/Prf/5-Dox-R: 2 µg/ml) or sorafenib (HepG2-Sora-R, Huh7-Sora-R, and Plc/Prf/5-Sora-R: 10 µM) for 24 h. Chemoresistance was confirmed by MTT assay.

#### Stable and transient H19 overexpression

Stable *H19* overexpression in hepatoma cells was established by transfection with a vector (pcDNa3.1(+)_A009) containing the synthetic *H19*-sequence or the empty vector as control (Figure S1) (Ref. No.: 1381790, Life Technologies, California, USA) using jetPEI^TM^ Hepatocyte reagent (102-05N, VWR International GmbH, Darmstadt, Germany) as recommended by the manufacturer. The final vector construct was verified by sequencing. Resistance to geneticin (G418, Invitrogen, Darmstadt, Germany) was conferred by the neomycin resistance gene (Neo (R)).

Transient *H19* overexpression in chemoresistant cells was performed accordingly, but cells were treated with doxorubicin or sorafenib (HepG2-Dox-R: 2 µg/ml; Huh7-Sora-R: 2.5 µM) simultaneously with the plasmid transfection. *H19* overexpression was confirmed by qPCR.

#### H19 knockdown

*H19* knockdown was performed in 96-well plates with antisense LNATM gapmer (Exiqon, Vedbaek, Denmark) for *H19* (5’-GACTTAGTGCAAATTA-3’) or negative control A (5’-AACACGTCTATACGC-3’) (gapmer concentration per well: HepG2: 0.04 µM, Plc/Prf/5: 0.02 µM, and Huh7: 0.03 µM) using INTERFERin® (Polyplus-Transfection, Illkirch, France) transfection reagent as recommended by the manufacturer. The negative control shows no homology to any known microRNA, lncRNA, or mRNA. *H19* knockdown was confirmed by qPCR.

### Cytotoxicity assay (MTT assay)

Hepatoma cells were seeded into 96-well plates and treated with different concentrations of doxorubicin (Sigma-Aldrich, Taufkirchen, Germany), sorafenib (Biomol GmbH, Hamburg, Germany), or the respective solvent control. 24 h after treatment, medium was removed and 0.5 mg/ml MTT (3-[4,5-dimethylthiazol-2-yl]-2,5- diphenyltetrazolium bromide; thiazolyl blue) (Sigma-Aldrich, Taufkirchen, Germany) diluted in medium was added. After 2 h incubation, the formazan crystals were dissolved in dimethyl sulfoxide, and the absorbance was measured at 550 nm with 630 nm as reference wavelength in a microplate reader (Tecan Sunrise™, Tecan Group Ltd., Männedorf, Switzerland) 64,65.

For the MTT assay after 5-azacytidine treatment, cells were treated over four days with 2 µM of the DNA-methyltransferases inhibitor 5-azacytidine (Sigma-Aldrich, Taufkirchen, Germany), which was freshly added each day.

### Clonogenicity assay

The hepatoma cells HepG2, Huh7, and Plc/Prf/5 were seeded into 6-well plates, allowed to attach overnight, and treated with the indicated concentrations of sorafenib or doxorubicin for another 24 h. Following the treatment, cells were washed with PBS and allowed to form colonies in complete growth medium. After 10 to 15 days, the colonies were fixed in methanol, stained with crystal violet, and counted. For the sorafenib experiments colonies were counted with a clono counter software as previously described 66,67 and for the doxorubicin experiments colonies were counted manually.

### Ki67 staining

Cells were detached from the plates using trypsin (Sigma-Aldrich, Taufkirchen, Germany) and cell staining was performed as described previously 51. For intracellular staining of Ki67, cells were washed with flow cytometry buffer (FCB; PBS containing 2.5% (v/v) bovine calf serum and 0.05% (w/v) NaN3) and fixed for 10 min in 1% (w/v) paraformaldehyde in PBS, pH 7.6, followed by permeabilization in SAP (FCB with 0.2% (w/v) saponin, and blocking for 30 min in 20% FCS (v/v, diluted in SAP). Cells were incubated with Ki67 or isotype control antibody (10 µl in 50 μl of FCB; BD Biosciences Heidelberg, Germany) for 15 min on ice. The cells were washed in FCB and resuspended in 1% (w/v) cold paraformaldehyde in PBS, pH 7.6. The stained cells were examined on a BD LSRFortessa™ cell analyser and results were analyzed using the FACS Diva software (BD Biosciences, Heidelberg, Germany).

### DNA methylation analysis

#### DNA extraction and bisulfite conversion

Genomic DNA from HepG2, Plc/Prf/5, and Huh7 cells was extracted with the GenEluteTM Mammalian Genomic DNA Miniprep Kit (Sigma-Aldrich, Taufkirchen, Germany) and bisulfite treatment of 500 ng genomic DNA was performed with the EZ DNA Methylation-Gold Kit (Zymo Research, Freiburg, Germany) according to the manufacturer’s instructions.

#### Single nucleotide primer extension (SNuPE)

Amplicons were generated using a previously described protocol 68 and region-specific primers for the *H19* promoter (10 μM each): forward (5’-3’): GGGTTTGGGAGAGTTTGTGAGGT; reverse (5’-3’): AACACAAAAAACCCCTTCCTACCA. The PCR reaction conditions were 15 min at 95°C followed by 42 cycles of 95°C for 60 s, 57.6°C for 60 s, 72°C for 60 s, and a final 5 min extension at 72°C. The single nucleotide primer extension for two CpG sites of the *H19* promoter (CpG 3 primer (5’-3’): TGTTAGTAGAGTG and CpG 17 primer (5’-3’): GTGATTAGTATAAGTT) was performed as previously described 42.

#### Local deep bisulfite sequencing (Bi-PROF)

For the analysis with next generation sequencing, the recommended adaptors were added to the primer sequences for the amplicon generation (forward (5’-3’): TCTTTCCCTACACGACGCTCTTCCGATCTGGGTTTGGGAGAGTTTGTGAGGT and reverse (5’-3’): GTGACTGGAGTTCAGACGTGTGCTCTTCCGATCTAACACAAAAAACC CCTTCCTACCA, annealing temperature 60°C). Purified PCR products were pooled in an equimolar ratio and sequenced 69 on a MiSeq instrument with the sequencing-by-synthesis technology (2 x 300 bp paired-end) aiming at 10,000 reads per amplicon according to the manufacturer’s protocol.

### DEN mouse model

All animal procedures were performed in accordance with the local animal welfare committee (36/2013, 36/2014). Mice were kept under controlled conditions regarding temperature, humidity, 12 h day/night rhythm, and food access.

For the long-term experiment, *H19* knockout (*H19*Δ3; H19 ko) mice 48,70 matched with wild-type (wt) littermates were intraperitoneally injected with 5 mg/kg body weight diethylnitrosamine (DEN) (Sigma-Aldrich, Taufkirchen, Germany) at the age of two weeks and sacrificed 24 weeks after injection. Untreated mice served as control (co) (female: *H19* wt co n=11, *H19* ko co n=23, *H19* wt DEN n=22, *H19* ko DEN n=25; male *H19* wt co n=13, *H19* ko co n=18, *H19* wt DEN n=25, *H19* ko DEN n=20) 71,72.

For the short-term experiment nine week old male mice were intraperitoneally injected with either 100 mg/kg body weight DEN or NaCl as a sham-control (each, n=5). Mice were sacrificed 48 h after the injection 72,73,74.

### Immunohistochemistry

Hematoxylin-eosin staining and immunohistological staining for the proliferation marker Ki67 on paraffin-embedded tissues and sample examination were performed as previously described 19,72,75.

### Chromogenic *in situ *hybridization (CISH)

Eight paraffin-embedded samples from HCC patients were investigated. The reseach project was authorized by the ethical committee of the Medical University of Graz (Ref. Nr. 20-119 ex 08/09). CISH was performed using the miCURY LNATM microRNA ISH Optimization Kit (FFPE) (Exiqon, Vedbaek, Denmark) according to manufacturer’s instruction. A biotin-labeled probe was used for the detection of *H19* RNA (/5BioTEG/GTCCTGTAACCAAAAGTGACCG, Exiqon, Vedbaek, Denmark). A digoxin-labeled probe of scrambled RNA served as negative control (/5DigN/GTGTAACACGTCTATACGCCCA, Exiqon, Vedbaek, Denmark) and a digoxin-labeled beta-actin probe was used as positive control (/5DigN/CTCATTGTAGAAGGTGTGGTGCCA, Exiqon, Vedbaek, Denmark). All probes were used in a concentration of 40 nM. Proteinase K digestion was done for 10 min at 37°C with 15 µg/ml Proteinase K (Roche, Mannheim, Germany). The hybridization step was performed at 56°C for 1 h in a slide hybridizer DakoCytomation (Dako, Hamburg, Germany). Nuclei were counterstained with Nuclear Fast Red Counterstain (Vector Laboratories, Burlingname, CA, USA).

### Statistical analysis

Data analysis and statistics were performed with Excel 2013 and OriginPro 8.6G (OriginLab Corporation, Northampton, USA). Values were expressed as mean ± SEM or as box plots with 25th/75th percentile boxes, geometric medians (line), means (square), and 10th/90th percentile as whiskers. Statistical differences were calculated using an independent two-sample t-test, Mann-Whitney U test, or Kolmogorov-Smirnov test as indicated depending on whether the data were normally distributed. A chi-square test was used for the statistical analysis of tumor development and characterization in mouse livers.

## SUPPLEMENTAL MATERIAL

Click here for supplemental data file.

All supplemental data for this article are also available online at http://www.cell-stress.com/researcharticles/the-long-non-coding-rna-h19-suppresses-carciogenesis-and-chemoresistcance-in-hepatocellular-carcinoma/.

## Abbreviations

DEN: diethylnitrosamine
HCC: hepatocellular carcinoma
IGF: insulin-like growth factor
lncRNA: long non-coding RNA
LOI: loss of imprinting
