# Supplementary material for: The long non-coding RNA H19 suppresses carcinogenesis and chemoresistance in hepatocellular carcinoma
Source: Cell Stress. 2017 Aug 25;1(1):37–54. doi: 10.15698/cst2017.10.105 (PMC6551655; doi:10.15698/cst2017.10.105)
Supplement: Supplementary file 1 [file ces-01-037-s01.pdf]

THE LONG NON-CODING RNA *H19* SUPPRESSES CARCINOGENESIS AND CHEMORESISTANCE IN HEPATOCELLULAR CARCINOMA

Christina S. Schultheiss, Stephan Laggai, Beate Czepukojc, Usama K. Hussein, Markus List, Ahmad Barghash, Sascha Tierling, Kevan Hosseini, Nicole Golob-Schwarzl, Juliane Pokorny, Nina Hachenthal, Marcel Schulz, Volkhard Helms, Jörn Walter, Vincent Zimmer, Frank Lammert, Rainer M. Bohle, Luisa Dandolo, Johannes Haybaeck, Alexandra K. Kiemer, Sonja M. Kessler

Supplementary information

Supplemental Table S1: Target gene-specific primer information
Supplemental Figure S1: Plasmid map and H19-sequence
Supplemental Figure S2: H19 expression in lasermicrodissected hepatocytes
Supplemental Figure S3: Effect of *H19* overexpression on colony formation ability

SUPPLEMENT

Supplemental Table S1: Target gene-specific primer information

**mRNA**	**forward primer sequence 5' → 3'**	**reverse primer sequence 5' → 3'**	**gene bank accession no.**	**AT [°C]**	**product size [bp]**	**primer concen-tration [µM]**
hu ABCB1/ hu MDR1	GCTATAATGCGA CAGGAGATAGGCT	CATTCCAATTTTGT CACCAATAACTT	NM_001348946.1;NM_001348945.1;NM_001348944.1	56	116	0.2
hu ACTB	TGCGTGACATTAA GGAGAAG	GTCAGGCAGCTC GTAGCTCT	NM_001101.3	60	107	0.2
hu ELAVL1	GGTGACATCGGG AGAACGAA	CCAAGCTGTGTCC TGCTACT	NM_001419.2	60	142	0.2
hu H19	TTCAAAGCCTCCA CGACTCT	CTGAGACTCAAGG CCGTCTC	NR_131224.1;NR_131223.1;NR_002196.2;NM_001293171.2	60	100	0.2
hu H19 IP	GCTCCCAGAACCC ACAACAT	CCTTCCAGAGCCG ATTCCTG	NR_131224.1;NR_131223.1;NR_002196.2;NM_001293171.2	61	149	0.2
hu IGF2	GGACTTGAGTCCC TGAACCA	TGAAAATTCCCGT GAGAAGG	NM_000612.5;NM_001007139.5;NM_001127598.2;NM_001291861.2;NM_001291862.2	56	100	0.25
mu H19	CAGAGGTGGATGT GCCTGCC	CGGACCATGTCAT GTCTTTCTGTC	NR_001592.1	60	80	0.25
mu Igf2	GGAAGTCGATGTT GGTGCTTCTC	CGAACAGACAAAC TGAAGCGTGT	NM_010514.3	60	121	0.2
mu Csnk2a2	GTAAAGGACCCTG TGTCAAAGA	GTCAGGATCTGGT AGAGTTGCT	NM_009974.3	60	85	0.4
**miRNA**	**forward primer sequence 5' → 3'**	**reverse primer sequence 5' → 3'**	**accession no.**	**AT [°C]**		**primer concen-tration [µM]**
hsa-miR-675	TGGTGCGGAAAG GGCCCACAGT	GAATCGAGCACCA GTTACGCAT	MIMAT0004284	64		0.2
RNU6B	ACGCAAATTCGTG AAGCGTT	GAATCGAGCACCA GTTACG	e.g. NR_125730.1	55		0.5

A

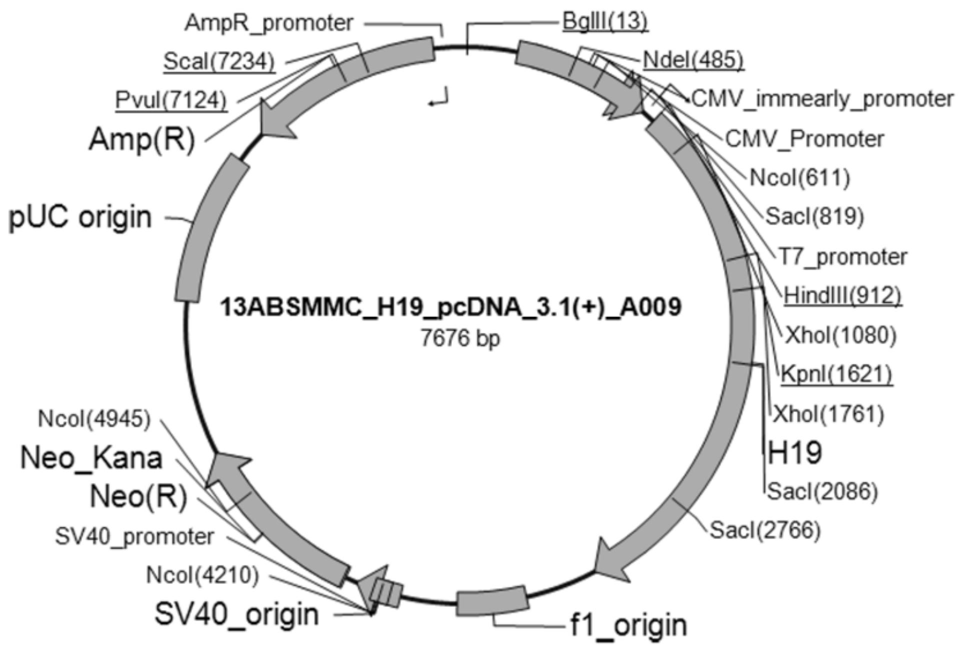

B

AAGCTTGGGAGGGGGTGGGATGGGTGGGGGGTAACGGGGGAAACTGGGGAAGTGGGGAACCGAGGGGCAACCAGG
GGAAGATGGGGTGCTGGAGGAGAGCTTGTGGGAGCCAAGGAGCACCTTGGACATCTGGAGTCTGGCAGGAGTGATGA
CGGGTGGAGGGGCTAGCTCGAGGCAGGGCTGGTGGGGCCTGAGGCCAGTGAGGAGTGTGGAGTAGGCGCCCAGGCA
TCGTGCAGACAGGGCGACATCAGCTGGGGACGATGGGCCTGAGCTAGGGCTGGAAAGAAGGGGGAGCCAGGCATTCA
TCCCGGTCACTTTTGGTTACAGGACGTGGCAGCTGGTTGGACGAGGGGAGCTGGTGGGCAGGGTTTGATCCCAGGGC
CTGGGCAACGGAGGTGTAGCTGGCAGCAGCGGGCAGGTGAGGACCCCATCTGCCGGGCAGGTGAGTCCCTTCCCTCC
CCAGGCCTCGCTTCCCCAGCCTTCTGAAAGAAGGAGGTTTAGGGGATCGAGGGCTGGCGGGGAGAAGCAGACACCCT
CCCAGCAGAGGGGCAGGATGGGGGCAGGAGAGTTAGCAAAGGTGACATCTTCTCGGGGGGAGCCGAGACTGCGCAA
GGCTGGGGGGTTATGGGCCCGTTCCAGGCAGAAAGAGCAAGAGGGCAGGGAGGGAGCACAGGGGTGGCCAGCGTAG
GGTCCAGCACGTGGGGTGGTACCCCAGGCCTGGGTCAGACAGGGACATGGCAGGGGACACAGGACAGAGGGGTCCC
CAGCTGCCACCTCACCCACCGCAATTCATTTAGTAGCAGGCACAGGGGCAGCTCCGGCACGGCTTTCTCAGGCCTATG
CCGGAGCCTCGAGGGCTGGAGAGCGGGAAGACAGGCAGTGCTCGGGGAGTTGCAGCAGGACGTCACCAGGAGGGCG
AAGCGGCCACGGGAGGGGGGCCCCGGGACATTGCGCAGCAAGGAGGCTGCAGGGGCTCGGCCTGCGGGCGCCGGT
CCCACGAGGCACTGCGGCCCAGGGTCTGGTGCGGAGAGGGCCCACAGTGGACTTGGTGACGCTGTATGCCCTCACCG
CTCAGCCCCTGGGGCTGGCTTGGCAGACAGTACAGCATCCAGGGGAGTCAAGGGCATGGGGCGAGACCAGACTAGGC
GAGGCGGGCGGGGCGGAGTGAATGAGCTCTCAGGAGGGAGGATGGTGCAGGCAGGGGTGAGGAGCGCAGCGGGCG
GCGAGCGGGAGGCACTGGCCTCCAGAGCCCGTGGCCAAGGCGGGCCTCGCGGGCGGCGACGGAGCCGGGATCGGT
GCCTCAGCGTTCGGGCTGGAGACGAGGCCAGGTCTCCAGCTGGGGTGGACGTGCCCACCAGCTGCCGAAGGCCAAG
ACGCCAGGTCCGGTGGACGTGACAAGCAGGACATGACATGGTCCGGTGTGACGGCGAGGACAGAGGAGGCGCGTCC
GGCCTTCCTGAACACCTTAGGCTGGTGGGGCTGCGGCAAGAAGCGGGTCTGTTTCTTTACTTCCTCCACGGAGTCGGC
ACACTATGGCTGCCCTCTGGGCTCCCAGAACCCACAACATGAAAGAAATGGTGCTACCCAGCTCAAGCCTGGGCCTTTG
AATCCGGACACAAAACCCTCTAGCTTGGAAATGAATATGCTGCACTTTACAACCACTGCACTACCTGACTCAGGAATCGG
CTCTGGAAGGTGAAGCTAGAGGAACCAGACCTCATCAGCCCAACATCAAAGACACCATCGGAACAGCAGCGCCCGCAG
CACCCACCCCGCACCGGCGACTCCATCTTCATGGCCACCCCCTGCGGCGGACGGTTGACCACCAGCCACCACATCATC
CCAGAGCTGAGCTCCTCCAGCGGGATGACGCCGTCCCCACCACCTCCCTCTTCTTCTTTTTCATCCTTCTGTCTCTTTGT
TTCTGAGCTTTCCTGTCTTTCCTTTTTTCTGAGAGATTCAAAGCCTCCACGACTCTGTTTCCCCCGTCCCTTCTGAATTTA

ATTTGCACTAAGTCATTTGCACTGGTTGGAGTTGTGGAGACGGCCTTGAGTCTCAGTACGAGTGTGCGTGAGTGTGAGC
CACCTTGGCAAGTGCCTGTGCAGGGCCCGGCCGCCCTCCATCTGGGCCGGGTGACTGGGCGCCGGCTGTGTGCCCG
AGGCCTCACCCTGCCCTCGCCTAGTCTGGAAGCTCCGACCGACATCACGGAGCAGCCTTCAAGCATTCCATTACGCCC
CATCTCGCTCTGTGCCCCTCCCCACCAGGGCTTCAGCAGGAGCCCTGGACTCATCATCAATAAACACTGTTACAGCAAA
AAAAAAAAAAAAATCTAGA

Supplemental Figure S1: (A) Plasmid map (pcDNa3.1(+)_A009) and (B) *H19*-sequence (2334 bp).

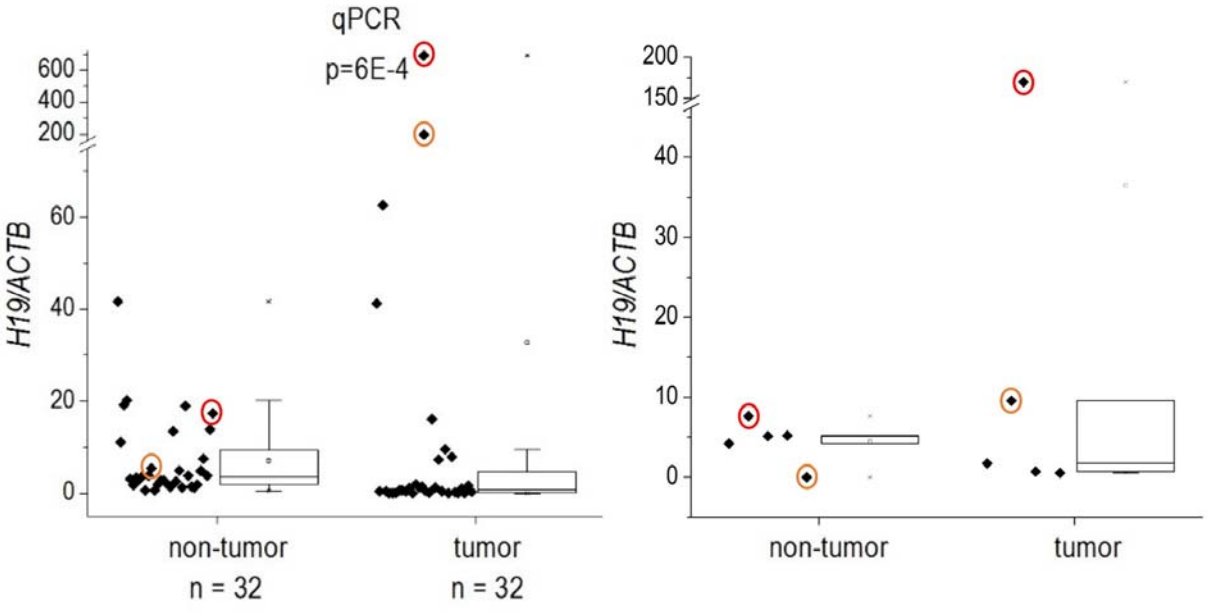

Supplemental Figure S2: H19 expression in HCC tissues from Saarland University Medical Center determined by qPCR (each, n=32, Mann-Whitney U test; see Figure 1D). Highlighted are the two highest expressing HCC tissues and corresponding normal tissues, from which hepatocytes were microdissected and compared with hepatocytes from three low expressing HCC tissues.

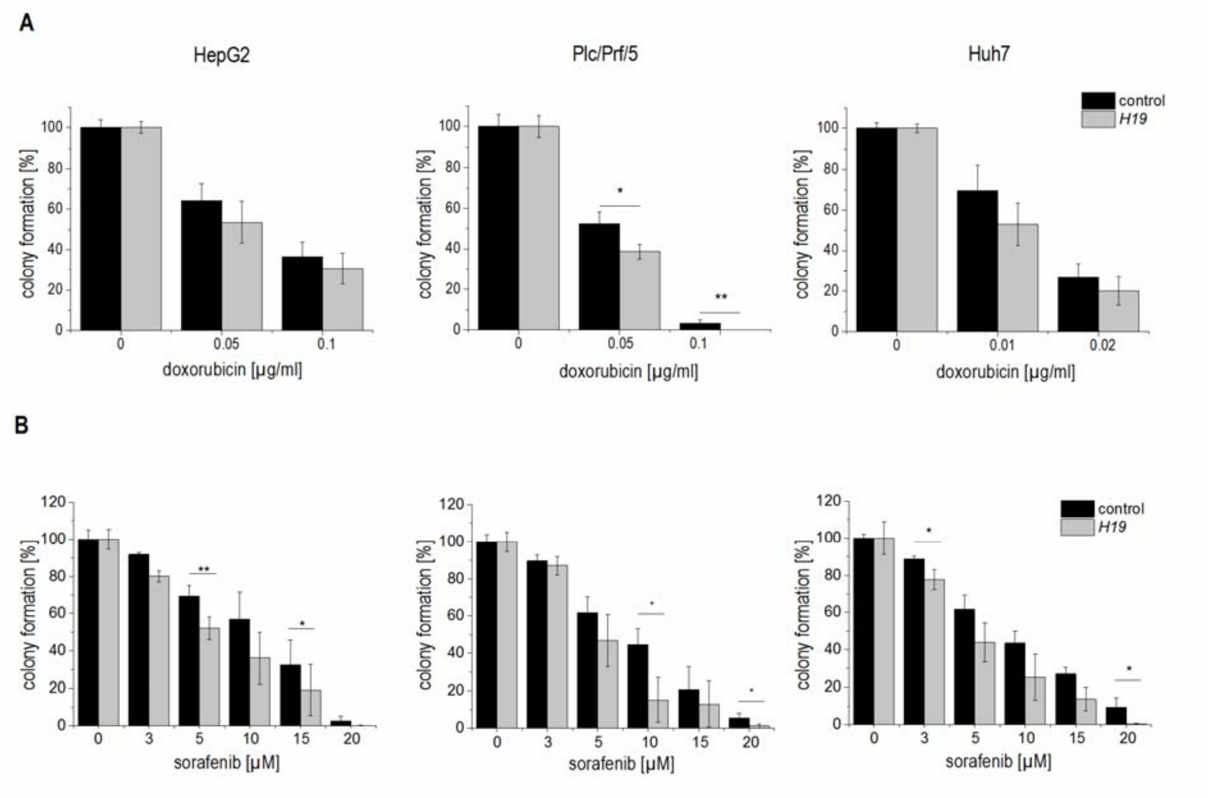

Supplemental Figure S3: Effect of *H19* overexpression on colony formation ability in stably *H19* overexpressing (*H19*) and vector control (control, co) HepG2 (left panels), Plc/Prf/5 (middle panels), and Huh7 (right panels) cells. (A, B) Colony formation ability of control or *H19* cells normalized to their respective untreated controls after (A) doxorubicin (n≥3, duplicates) or (B) sorafenib (n=3, triplicates) treatment. The p values were calculated by two-sample t-test or Mann-Whitney *U* test depending on the data distribution. * $p < 0.05$, ** $p < 0.01$, *** $p < 0.001$.
